# Traitement chirurgical d’un lymphœdème scrotal

**DOI:** 10.11604/pamj.2019.33.88.13066

**Published:** 2019-06-06

**Authors:** Adil Dehhaze, Yassine Benchamkha, Ouafa Dhaidah, Mouna Ejjiyar, Mariam Quaboul, Abdelkoddous Bhihi, Mehdi Sahibi, Moulay Driss Elamrani, Saloua Ettalbi

**Affiliations:** 1 Service de Chirurgie Plastique, Réparatrice, Esthétique et Brûlés, CHU Mohammed VI, Marrakech, Maroc

**Keywords:** Lymphœdeme, elephantiasis, reconstruction, Lymphoedema, elephantiasis, reconstruction

## Abstract

L’éléphantiasis scrotal se définit comme étant une augmentation du volume scrotal qui peut atteindre une taille très importante; il s’agit du patient O.H âgé de 70 ans, marié et père de 4 enfants, originaire et résident à Agadir (sud du Maroc), agriculteur de profession. Le début de la symptomatologie remonte à 7 ans par l’installation de l’œdème scrotal puis des deux pieds et jambes augmentant progressivement de volume. L’examen local mettait en évidence un éléphantiasis scrotal de 80cm de circonférence et des deux jambes et pieds. Une imagerie à résonnance magnétique (IRM) pelvienne a été demandée dans le cadre du bilan d’exploration locorégionale. Le patient a été programmé pour un geste d’exérèse scrotale en mono- bloc sous rachis anesthésie avec libération des deux testicules couvertes directement par la peau restante et de la verge qui a été couverte par greffe de peau mince. Le but du traitement est assurer la fonction et prendre en charge les préjudices esthétiques. Le traitement conservateur consistant à la dérivation lymphatique vers le réseau veineux ou à la dilatation des vaisseaux lymphatiques a été abandonné. On se base pour la prise en charge sur la chirurgie qui peut être abstenu en cas de contre indication absolue au geste chirurgical.

## Abstract (en)

Scrotal elephantiasis is defined as an increase in the scrotal volume which can reach a very large size. This study involved O.H, a married man aged 70 years, father of 4 children, farmer, native of and resident in Agadir (south of Morocco). Symptoms started 7 years before when scrotal edema and then an edema in both feet and legs gradually increasing in volume occurred. Physical examination showed scrotal elephantiasis with a circumference measured 80 cm and elephantiasis in both legs and feet. Locoregional assessment was based on pelvic magnetic resonance imaging (MRI). The patient was scheduled for monoblock scrotal resection under spinal anesthesia with removal of all of the tissue affected by the lymphedema around the testicle which were covered using the remaining skin and of the penis which was skin grafted using thin skin. The treatment aimed to ensure penis function and to manage the disfiguration. Conservative treatment based on lymphovenous bypass surgery or on the dilation of lymph vessels is no longer performed. Treatment is based on surgery. Surgery is avoided when there are absolute contraindications.

## Introduction

L’éléphantiasis scrotal se définit comme étant une augmentation du volume scrotal qui peut atteindre une taille très importante. Il a un retentissement fonctionnel (enfouissement de la verge), esthétique ainsi que psychologique. C’est une pathologie rare d’origine primitive (congénitale) ou secondaire (acquise). Son diagnostic repose sur la clinique et son traitement relève de la chirurgie. Nous reportons un cas d’éléphantiasis scrotal idiopathique pris en charge au sein du Service de Chirurgie Plastique au CHU de Marrakech avec une revue de littérature.

## Patient et observation

**Figure 1:**
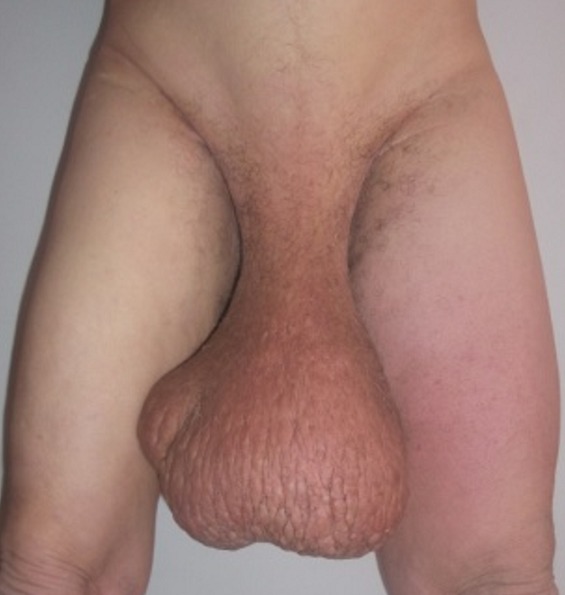
Vue de face en préopératoire: la bourse scrotale atteint les genoux vue son poids

**Figure 2:**
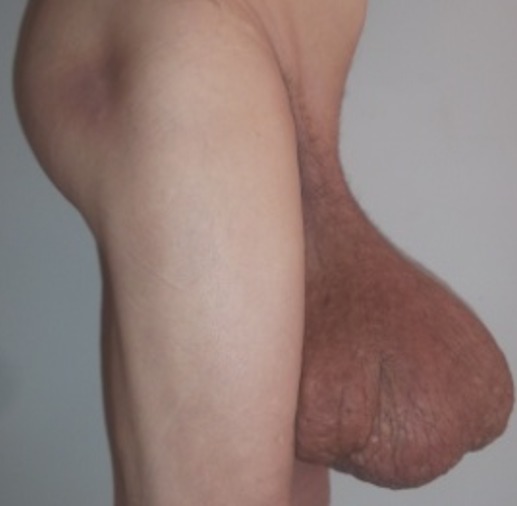
Vue de profil montrant le volume important de la bourse scrotale

Il s’agit du patient O.H âgé de 70 ans, marié et père de 4 enfants, originaire et résident à Agadir (sud du Maroc), agriculteur de profession, de bas niveau socio-économique. Opéré en 1996 pour kyste hydatique du foie, en 1997 pour une hernie sus ombilicale puis en 2009 pour grosse bourse. Il se présente pour un lymphœdème scrotal et des deux membres inférieurs. Le début de la symptomatologie remonte à 7 ans par l’installation de l’œdème scrotal puis des deux pieds et jambes augmentant progressivement de volume jusqu’à disparition de la verge entourée par la peau infiltrée le conduisant à consulter. Par ailleurs, le patient présente des troubles mictionnels à type de pollakiurie et dysurie. Le tout évoluant dans un contexte d’apyrexie et conservation de l’état général (Figure 1). L’examen général du patient ne montrait pas de particularité (TA = 13/4 cmhg, FC = 70 bpm). L’examen local mettait en évidence un éléphantiasis scrotal de 80 cm de circonférence et des deux jambes et pieds (Figure 2).

**Figure 3:**
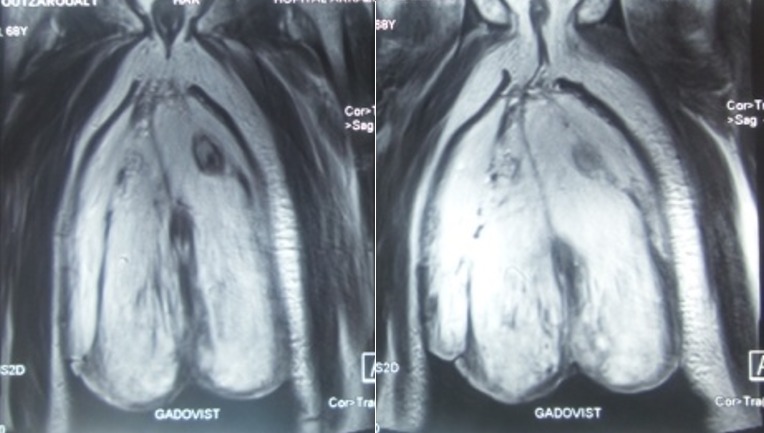
L’Imagerie à résonnance magnétique (IRM) pelvienne montrant les structures anatomiques de la bourse noyées dans l’œdème visualisé en hypersignal avec allongement du cordon spermatique et hypotrophie scrotale bilatérale

La peau en regard est très épaisse et le gland et la verge sont invisibles. Le toucher rectal ne mettait pas en évidence d’hypertrophie prostatique. L’examen des aires ganglionnaires ne montrait pas de particularité. Le reste de l’examen clinique notamment cardiaque et pleuro-pulmonaire était sans particularité à part une hernie sus ombilicale simple (récidive). Une IRM pelvienne a été demandée dans le cadre du bilan d’exploration locorégionale ayant montré une hydrocèle de grande abondance avec infiltration œdémateuse diffuse et épaississement des membranes scrotales sans masse tissulaire décelable. Les deux testicules sont atrophiques et refoulées en dehors avec un uretère allongé (Figure 3). Le doppler des deux membres inférieurs était normal ainsi que l’écho-cœur avec une fonction cardiaque respectée.

**Figure 4:**
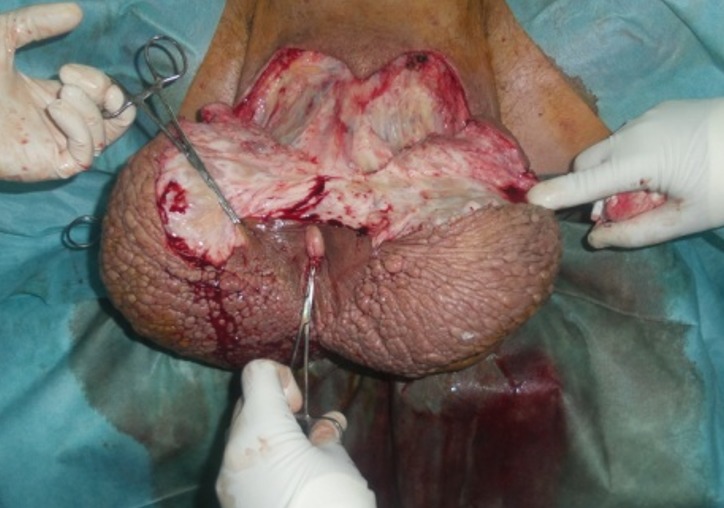
Vue peropératoire montrant l’abord chirurgical en y permettant d’avoir un bon jour pour disséquer la verge et les testicules

**Figure 5:**
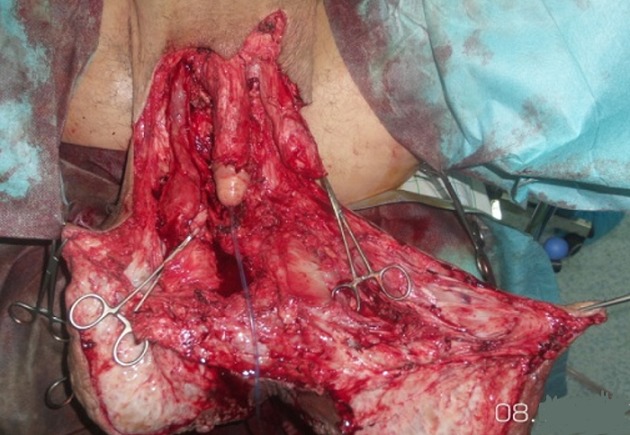
Vue peropératoire montrant la libération de la verge et des deux testicules

**Figure 6:**
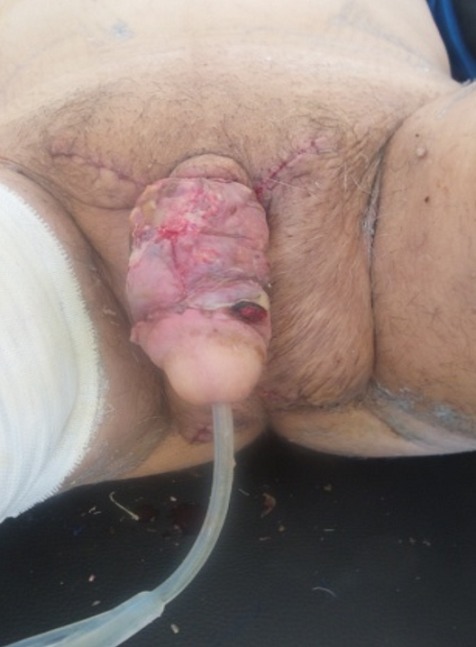
Photo postopératoire à 9 jours avec un processus cicatriciel normal, sonde urinaire en place

**Figure 7:**
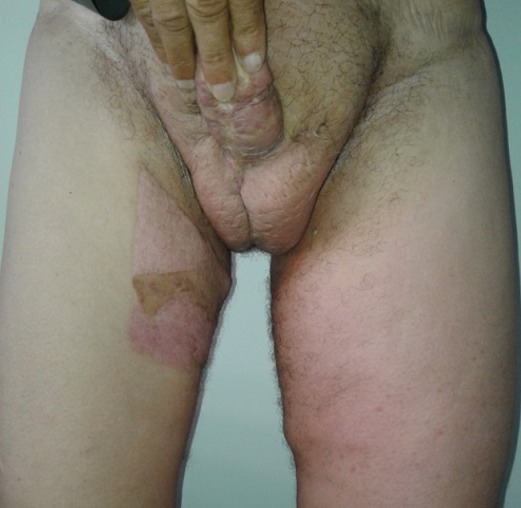
Photo postopératoire de 6 mois avec une bonne cicatrisation et reprise de la fonction et du confort du patient

Un bilan biologique montrait une Hb = 11,7 g/dl; GB = 11750 et Pq = 169 000. Urée = 0,39 g/l; créatinine = 8,7 mg/l; glycémie = 1 g/l et un bilan d’hémostase normal. Le patient a été programmé pour un geste d’exérèse scrotale en mono- bloc sous rachis anesthésie avec libération des deux testicules couvertes directement par la peau restante et de la verge qui a été couverte par greffe de peau mince prélevée de la face interne de la cuisse droite. Par ailleurs, il avait une sténose de l’urètre antérieur qui a été dilatée avec mise en place d’une sonde vésicale siliconée N°18 (Figure 4 et Figure 5). La pièce opératoire pesait 8kg et elle a été adressée à l’anatomopathologiste. Les suites post opératoires étaient simples. L’ablation des fils était faite à J10 post opératoire avec la greffe qui a tenu à 80% (Figure 6). Le résultat anatomopathologique a montré qu’il s’agit d’un remaniement inflammatoire aigue et chronique sans signe de malignité ou de spécificité (Figure 7).

## Discussion

Le lymphœdème scrotal est caractérisé par la présence d’un épanchement liquidien riche en protéines au niveau scrotal et pénien. Il affecte souvent le sexe masculin. Les membres inférieurs peuvent être atteints dans certains cas [1]. L’étiologie peut être primitive (idiopathique) du fait d’une dysplasie congénitale irréversible du système lymphatique scrotal [2, 3]. La composition des sols a été incriminée, surtout les sols volcaniques riches en silicate d’aluminium qui serait absorbé par voie transdermique, par le contact des pieds avec le sol argileux. Elle peut être secondaire (acquise) à une infection parasitaire (filariose) dans les pays d’endémie filarienne, induisant une obstruction canalaire. A l’obstruction lymphatique intrinsèque ou extrinsèque par: une chirurgie carcinologique abdominale ou pelvienne, radiothérapie, affection inflammatoire chronique ou après une stase veineuse chronique [4, 5].

Le sarcome de kaposi ainsi que certaines infections vénériennes chroniques ont étés décrites comme causes de certains cas d’éléphantiasis [6]. Une dialyse péritonéale ambulatoire continue ou un ancien traumatisme peuvent aussi être la cause de lymphœdème scrotal. Cliniquement, l’atteinte péno-scrotale est la plus fréquente avec un scrotum volumineux pouvant atteindre des dimensions importantes induisant l’enfouissement de la verge comme c’était le cas de notre patient. Le retentissement de ce dernier est significatif sur la fonction mictionnelle induisant des infections urinaires, un rétrécissement urétral allant parfois jusqu’à la dilatation pyelo-calicielle; ainsi que sur la vie sexuelle du patient [7]. Le délai entre le début de la symptomatologie et la première consultation est le plus souvent long jusqu’à ce que la bourse atteigne un volume important comme c’est le cas de notre patient [8]. Paracliniquement, l’échographie et le scanner déterminent une origine obstructive extrinsèque des vaisseaux. Le doppler vasculaire montre la dilatation des lymphatiques et en cas de filariose objectivant les parasites. Cet examen n’a pas montré de particularité chez notre patient. D’autres examens biologiques peuvent être utilisés à la recherche de chlamydiae ou des micros filaires dans le sang [9].

Sur le plan thérapeutique: le but du traitement est assurer la fonction et prendre en charge les préjudices esthétiques. Le traitement conservateur consistant à la dérivation lymphatique vers le réseau veineux ou à la dilatation des vaisseaux lymphatiques a été abandonné. On se base pour la prise en charge sur la chirurgie qui peut être abstenu en cas de contre indication absolue au geste chirurgical. Il consiste à la résection large des tissus infiltrés par l’œdème. La cicatrisation peut être suivie en dirigé mais elle est lente et prolonge le séjour hospitalier mais l’idéal c’est la couverture par les tissus restants associée ou non à une greffe de peau autologue, comme c’est le cas de notre patient, voir l’utilisation de lambeaux locaux [10]. L’évolution est le plus souvent satisfaisante mais il y a toujours un risque de récidive dû à une obstruction lymphatique persistante ou bien récidivante. La prévention consiste à protéger la porte d’entrée des infections par le lavage quotidien du membre atteint. Des manœuvres simples pour favoriser le drainage lymphatique peuvent être suivies se basant sur l’élévation des membres et l’exercice physique ainsi qu’un portage de chaussures adaptées [11].

## Conclusion

L’éléphantiasis scrotal est une pathologie qui reste rare en dehors des zones d’endémie filarienne. Ses étiologies restent différentes d’où l’intérêt d’un bon interrogatoire, examen clinique et une exploration paraclinique bien conduite. La prise en charge reste chirurgicale avec des résultats très satisfaisants. La prévention reste un moyen pour diminuer l’aggravation clinique.

## Conflits d’intérêts

Les auteurs ne déclarent aucun conflit d'intérêts.
